# Peri-apatite coating decreases uncemented tibial component migration: long-term RSA results of a randomized controlled trial and limitations of short-term results

**DOI:** 10.1080/17453674.2018.1469223

**Published:** 2018-05-09

**Authors:** Koen T Van Hamersveld, Perla J Marang-Van De Mheen, Rob G H H Nelissen, Sören Toksvig-Larsen

**Affiliations:** a Department of Orthopaedics;; b Medical Decision Making, Leiden University Medical Center, Leiden, The Netherlands;; c Department of Orthopaedics, Hässleholm Hospital, Hässleholm, Sweden and Department of Clinical Sciences, Lund University, Lund, Sweden

## Abstract

Background and purpose — Biological fixation of uncemented knee prostheses can be improved by applying hydroxyapatite coating around the porous surface via a solution deposition technique called Peri-Apatite (PA). The 2-year results of a randomized controlled trial, evaluating the effect of PA, revealed several components with continuous migration in the second postoperative year, particularly in the uncoated group. To evaluate whether absence of early stabilization is diagnostic of loosening, we now present long-term follow-up results.

Patients and methods — 60 patients were randomized to PA-coated or uncoated (porous only) total knee arthroplasty of which 58 were evaluated with radiostereometric analysis (RSA) performed at baseline, at 3 months postoperatively and at 1, 2, 5, 7, and 10 years. A linear mixed-effects model was used to analyze the repeated measurements.

Results — PA-coated components had a statistically significantly lower mean migration at 10 years of 0.94 mm (95% CI 0.72–1.2) compared with the uncoated group showing a mean migration of 1.72 mm (95% CI 1.4–2.1). Continuous migration in the second postoperative year was seen in 7 uncoated components and in 1 PA-coated component. All of these implants stabilized after 2 years except for 2 uncoated components.

Interpretation — Peri-apatite enhances stabilization of uncemented components. The number of components that stabilized after 2 years emphasizes the importance of longer follow-up to determine full stabilization and risk of loosening in uncemented components with biphasic migration profiles.

Early migration of tibial components, which can be accurately measured with radiostereometric analysis (RSA), has been shown to predict future aseptic loosening (Ryd et al. 1995, Pijls et al. 2012b). Uncemented components typically display a biphasic migration pattern with high initial migration before stabilization (Pijls et al. 2012a, Wilson et al. 2012, Henricson and Nilsson 2016), while cemented components are initially more stable as the cement provides instant fixation, yet continuous bone resorption at the cement–bone interface may result in continuous migration (Nilsson et al. 2006, van Hamersveld et al. 2017). Given the importance of stabilization in the first months after implantation, one method to improve bone ingrowth after uncemented total knee arthroplasty (TKA) is the application of osteoconductive hydroxyapatite (HA) coatings (Nelissen et al. 1998, Carlsson et al. 2005).

Most HA coatings are plasma sprayed onto the porous beaded implant surface area. Plasma spraying is a “line of sight” technique and therefore only able to coat the substrate surface (Hansson et al. 2008). Contrarily, Peri-Apatite HA (PA) (Stryker, Mahwah, NJ, USA)is an alternative technique to deposit HA from an aqueous solution at room temperature, thereby increasing the coverage of HA onto the 3D beaded implant surface (Serekian 2004). However, without the effect of high temperatures up to 20,000 °C associated with plasma spraying, the HA remains pure and 100% crystalline, while a lower crystallinity has been shown to improve the bioactivity and resorption profile of HA (Overgaard et al. 1999, Serekian 2004). In addition, the adhesion of the relatively thin PA layer (of 20 µm compared with 50–75 µm for most HA coatings) is fragile when touching the coated metal during implantation and, like any HA coating, might delaminate or release particles over time (Bloebaum et al. 1994, Morscher et al. 1998). Only a few randomized RSA studies have assessed the short-term (2-year follow-up) effect of PA on uncemented tibial component migration (van der Linde et al. 2006, Hansson et al. 2008, Therbo et al. 2008, Molt and Toksvig-Larsen 2014). All trials concluded that the PA coating appears to improve stabilization up to 2 years after implantation. However, no studies have examined long-term migration profiles of PA-coated tibial components. It is therefore unknown whether the found short-term effect on component fixation is sustained over time. Furthermore, in the short-term report of the current study (Molt and Toksvig-Larsen 2014), a number of both uncoated and PA-coated components showed continuous migration in the second postoperative year. It is unclear whether this leads to future aseptic loosening or if this high initial migration is merely part of a migration pattern typical for uncemented components. We therefore now report 10-year follow-up results of this double-blinded, randomized controlled trial comparing implant migration measured with RSA and clinical results of PA-coated with uncoated uncemented TKAs.  

## Patients and methods

### Study design

Full details of the design and patient selection of this randomized controlled trial have been described previously (Molt and Toksvig-Larsen 2014). In short, all consecutive patients scheduled to undergo TKA due to primary osteoarthritis from July 2007 until February 2008 in Hässleholm Hospital (Sweden) were asked to participate. 60 patients were randomized in a 1:1 ratio. Patients received either “PA-coated” (applied on both the femoral and tibial component) or “uncoated” components of an otherwise identical (fully) uncemented cruciate retaining Triathlon total knee prosthesis (Stryker, Mahwah, NJ, USA). The porous undersurface (in both versions) consisted of cobalt-chromium sintered beads with a porosity of 35% and mean pore size of 425 µm. Highly cross-linked polyethylene inserts were used in all cases.

At all follow-up points, the Knee Society Score (KSS) and the Knee injury and Osteoarthritis Outcome Score (KOOS) were obtained. Both patients and observers performing clinical follow-up and RSA measurements remained blinded to the allocated group during the entire follow-up period.

### Radiostereometric analysis

RSA radiographs were made on the first day after surgery when weight bearing was achieved. Subsequent examinations were performed after 3 months, 1 year, 2, 5, 7, and 10 years. RSA radiographs were performed in supine position with the knee in a calibration cage (Cage 10, RSA Biomedical, Umeå, Sweden). RSA measurements were analyzed using UmRSA software (v6.0, RSA, Biomedical, Umeå, Sweden). Positive directions along and about the orthogonal axes are according to RSA guidelines (Valstar et al. 2005). Migration was described as translation of the geometric center of the prosthesis markers and rotation of the rigid body defined by the prosthesis markers about this geometric center of gravity. The length of the translation vector of the marker or virtual marker in a rigid body that has the greatest migration, i.e., the maximum total point motion (MTPM), was used as the primary outcome measure (ISO 16087:2013(E) 2013). The first postoperative RSA examination served as the reference for the migration measurements. Individual components with “continuous migration,” defined by Ryd et al. (1995) as an increase in MTPM of 0.2 mm or more in the second postoperative year, were classified as “loose.” This threshold was set at 0.1 mm per year after 2-year follow-up according to the modified continuous migration criterion (Ryd et al. 1995). Consequently, implants classified in the second postoperative year as loose were considered stabilized if the migration was less than 0.1 mm/year between 2-year and final follow-up (Wilson et al. 2012, Molt et al. 2016). The precision of the local RSA set-up after the 2-year follow-up period, specified as the 95% confidence interval (CI) around zero motion, and measured with 15 double examinations (ISO 16087:2013(E) 2013), was 0.10 mm, 0.10 mm, and 0.09 mm for transverse, longitudinal, and sagittal translations; 0.20°, 0.20°, and 0.24° for transverse, longitudinal, and sagittal rotations, respectively. The mean error of rigid body fitting of the RSA markers was below 0.35 mm and the upper limit for the condition number was set at 120, complying with the suggested limits of the RSA guidelines (ISO 16087:2013(E) 2013). The mean condition number was 40 (CI 37–42) and 51 (CI 49–54) for the implant and tibial markers, respectively.

### Statistics

Given the high accuracy of RSA measurements, only 17 patients were needed in each group to detect a decrease in migration from 1.0 to 0.5 ± 0.5 mm with 80% power and alpha set at 0.05, as described previously (Molt and Toksvig-Larsen 2014). 30 patients were randomized to each group to account for possible dropouts. The original primary outcome reported by Molt and Toksvig-Larsen (2014) was a difference in migration (MTPM) after 2 years of follow-up. For this long-term outcome report, the primary outcome was a difference in MTPM after 10 years of follow-up as registered at ClinicalTrials.gov (ID: NCT03198533). Data were analyzed according to the intention-to-treat principle. A linear mixed-effects model was used for all repeated measurements to effectively deal with missing values within patients during follow-up. As MTPM is always a positive vector, normal distribution was only obtained after log-transformation (logMTPM), computed as log10(MTPM +1). Differences in mean progression of logMTPM between groups were modeled as a function of time and the interaction of time with treatment. A random-intercepts term was used and remaining variability was modelled with a heterogeneous autoregressive order 1 covariance structure. Secondary outcomes (RSA translations and rotations, flexion, extension, KSS, and KOOS scores) were analyzed with a similar mixed-effects model. Differences in mean migration along and about each orthogonal axis were calculated using log-transformed absolute values (as the resultant of positive and negative displacement vectors requires all vectors to act on the same prosthesis) (Derbyshire et al. 2009). Given the non-normal distribution of knee extension and the KSS knee score (not resulting in a normal distribution after a log transformation), a comparable generalized estimating equations (GEE) approach was used to correct the standard errors via the sandwich estimator. Post hoc testing was performed to estimate between-group differences in MTPM using 3 months, 1 year, and 2 years as the reference. IBM SPSS Statistics 24.0 (IBM Corp, Armonk, NY, USA) was used for all outcome measures; a p-value <0.05 was considered significant.

### Ethics, registration, funding, and potential conflicts of interest

The trial was performed in compliance with the Declaration of Helsinki and Good Clinical Practice guidelines. This trial was approved by the local ethics committee prior to enrollment (entry no. 445/2005) and registered at ClinicalTrials.gov (new ID: NCT03198533, originally registered in 2007 as a sub-study of NCT00436982). Informed consent was obtained from all patients. Stryker provided funds in support of the costs associated with RSA radiographs and extra clinical follow-up examinations. The sponsor did not take any part in the design, conduct, analysis, and interpretations stated in the final manuscript.

## Results

**Figure 1.**
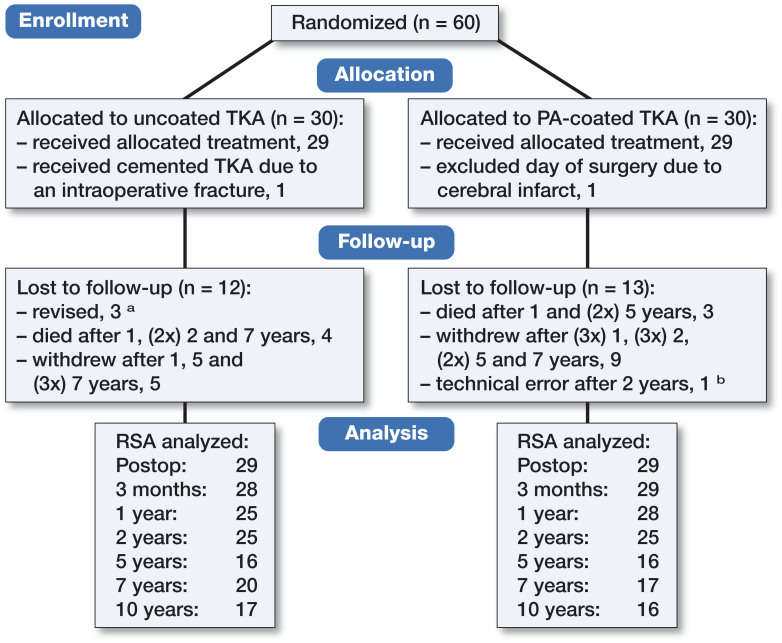
CONSORT flow diagram. TKA = total knee arthroplasty. **^a^** revised after 3 months (early infection), 1 year (late infection), and 10 years (mechanical failure). **^b^** clinical follow-up only, see text.

60 patients were randomized, of which 1 patient in each group was excluded on the day of surgery. Baseline characteristics were similar (Table 1). During follow-up, 3 knees were revised (2 infections and 1 loosening, see adverse events), 7 patients died, 14 patients refused further follow-up due to the burden of coming to the clinic at high age or moving out of the region, and 2 patients could not be analyzed reliably for technical reasons (Figure 1). Of the 2 cases with unreliable measurements, 1 had insufficient bone markers available causing high condition numbers (up to 216) after 1 year; reversed RSA migration results showed stable minor translations, and this patient had no knee complaints and no signs of loosening on conventional radiographs. The other case had unreliable measurements after 5 years (condition number of 135) due to over-projection of the femoral component and this component was revised after 10 years for mechanical failure (see below).

**Table 1.** Baseline demographic characteristics. Values are mean (SD) unless otherwise specified

	Uncoated	PA-coated
	(n = 29)	(n = 29)
Age	67 (6.8)	65 (8.1)
Body mass index	30 (4.3)	30 (4.9)
Female sex (n)	16	17
Previous knee surgery (n)		
none	22	25
joint debridement	1	1
meniscectomy	5	2
other	1	1
Ahlbäck’s grade (n)		
II	12	6
III	15	22
IV	2	1
ASA classification (n)		
I	8	6
II	20	21
III	1	2
Hip–knee–ankle angle		
preoperative	175 (5.0)	176 (6.2)
postoperative	179 (2.8)	179 (3.2)

### RSA migration measurements

**Figure 2.**
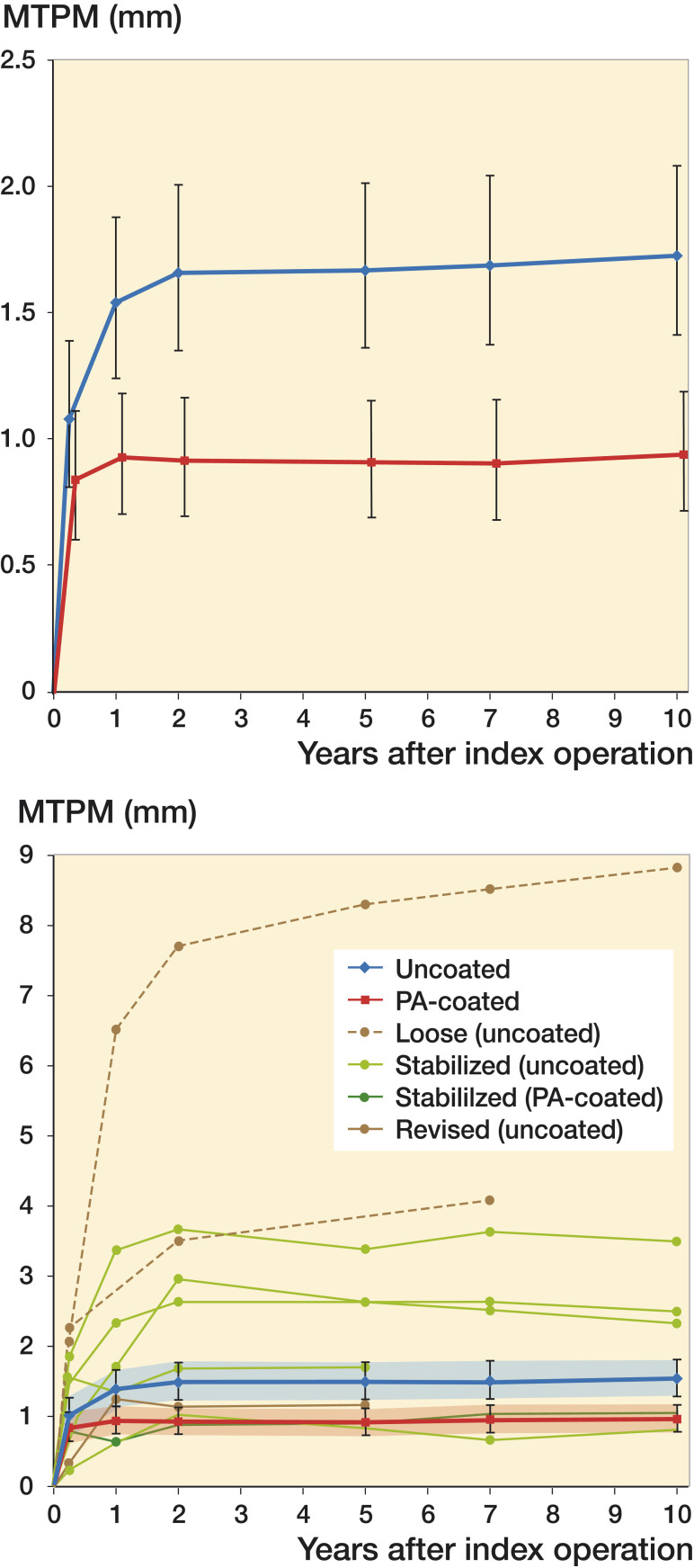
Maximum total point motion (back-transformed in the original scale in mm) during 10 years of follow-up: (top) the mean and 95% CI for the groups and (bottom) the mean and 95% CI for the groups and separate lines for the components showing continuous migration in the second postoperative year (in green the stabilized components after 2 years, in dashed brown the components failing to stabilize after 2 years and suspected for aseptic loosening, and in solid brown the revised component).

PA-coated components stabilized earlier as compared with uncoated components, resulting in a lower mean migration at 10 years: 0.94 mm (CI 0.72–1.2) for the PA-coated group and 1.7 mm (CI 1.4–2.1) for the uncoated group (p < 0.001). Over time, differences in migration between groups were seen in almost any direction (Table 2). Most of the difference in migration was already seen at 1 year, as the PA-coated components stabilized within the first 3 months while the uncoated components stabilized after 1 year of follow-up (Figure 2). Post hoc analysis showed that when using different baselines, no statistically significant between-group mean differences were seen from 1 year onwards (p = 0.1) and from 2 years onwards (p = 0.7) (Table 3, see Supplementary data).

**Table 2.** RSA migration measurements in absolute mm or degrees (95% CI) (log-transformed values are back-transformed in the original scale)

	1 year		2 years		10 years		
	Uncoated	PA-coated	Uncoated	PA-coated	Uncoated	PA-coated	p-value^a^
Translations (mm):							
Transverse	0.4 (0.28–0.49)	0.3 (0.21–0.40)	0.4 (0.33–0.54)	0.3 (0.19–0.38)	0.4 (0.30–0.54)	0.4 (0.24–0.48)	0.2
Longitudinal	0.5 (0.42–0.67)	0.3 (0.18–0.39)	0.5 (0.41–0.66)	0.3 (0.17–0.38)	0.5 (0.41–0.69)	0.3 (0.17–0.41)	< 0.001
Sagittal	0.5 (0.42–0.68)	0.3 (0.17–0.38)	0.7 (0.52–0.80)	0.2 (0.13–0.34)	0.7 (0.55–0.87)	0.3 (0.17–0.42)	< 0.001
Rotations (°):							
Transverse	1.1 (0.79–1.37)	0.6 (0.39–0.83)	1.2 (0.92–1.55)	0.6 (0.35–0.80)	1.3 (0.94–1.64)	0.6 (0.33–0.83)	< 0.001
Longitudinal	0.7 (0.50–0.82)	0.3 (0.17–0.42)	0.8 (0.67–1.04)	0.3 (0.18–0.44)	1.0 (0.78–1.21)	0.3 (0.14–0.43)	< 0.001
Sagittal	0.6 (0.49–0.83)	0.4 (0.30–0.59)	0.8 (0.66–1.04)	0.5 (0.36–0.67)	0.8 (0.57–0.97)	0.5 (0.30–0.65)	0.004
MTPM (mm)	1.5 (1.24–1.88)	0.9 (0.71–1.18)	1.7 (1.35–2.01)	0.9 (0.70–1.17)	1.7 (1.41–2.08)	0.9 (0.72–1.19)	< 0.001

a p-values stated in this column indicate testing the between-group mean differences with time over the entire postoperative follow-up period.

Between 1 and 2 years of follow-up, 7 uncoated components showed more than 0.2 mm MTPM and were suspected for loosening, compared with 1 in the PA-coated group. 5 of the 7 uncoated components stabilized, while 2 did not: 1 (clinically still asymptomatic patient) showed continuous migration of 0.14 mm/year up to 10-year follow-up (Figure 3, see Supplementary data) and 1 showed continuous migration of 0.11 mm/year up to 7-year follow-up who, despite having progressive complaints, refused to visit for 10-year follow-up (Figure 4, see Supplementary data). 1 uncoated component that was initially classified as loose was lost to follow-up but showed full stabilization at final (5-year) follow-up. 1 uncoated component was revised after 10 years as the patient had increasing pain and instability due to mechanical failure (see below). The PA-coated component initially classified as loose was stabilized at 5-year follow-up. None of the PA-coated components classified as stable showed continuous migration at any follow-up measurement beyond 2 years.

### Clinical results and adverse events

There were no statistically significant between-group differences with respect to improvement in knee flexion, extension, both KSS scores, and 4 of 5 KOOS subscales. The KOOS subscale quality of life improved equally between groups up to 5-year follow-up (p = 1.0), but substantially decreased in the PA-coated group between 5 and 10 years, resulting in a between-group mean difference after 10-year follow-up (p = 0.02) (Table 4, see Supplementary data).

3 patients (all with uncoated components) underwent revision surgery; the first due to an early prosthetic joint infection (at 3 months), the second due to a late infection (at 1 year) and the third (at 10 years) due to mechanical failure (complaints of pain and instability, posteromedial wear of the insert, and tibial component loosening was found during revision surgery) (Figure 5, see Supplementary data). 1 patient (randomized to the uncoated group) received a cemented implant due to an intraoperative fissure of the proximal tibia and was excluded. 1 patient (randomized to the PA-coated group) was transferred on the day of surgery to another hospital to receive appropriate treatment after a cerebral infarct and was also excluded.  

## Discussion

Our results show that the short-term effect of Peri-Apatite™ on uncemented tibial component migration is sustained over time, resulting in less mean migration and absence of components with continuous migration after 10 years. As shown in other long-term RSA studies, stabilization of uncemented tibial components can be achieved despite high initial migration (Pijls et al. 2012a, Henricson and Nilsson 2016). In the present long-term study, 6 individual components stabilized even after 2 years. Only 2 uncoated components migrated continuously throughout follow-up. Given that most prostheses stabilized within 2 years, the mean migration from 1 year onwards was not statistically significantly different between groups as confirmed in the post hoc analysis.

Both “excessive” initial migration in the first year (of more than 0.5 mm for a group of patients) and continuous migration after 1 year (0.2 mm in the second postoperative year for an individual patient) are associated with, and frequently used as predictors for, aseptic loosening (Ryd et al. 1995, Pijls et al. 2012b). These studies, however, combined prostheses that rely on primary fixation (cemented and uncemented with screws) and those that rely on secondary biological fixation (uncemented) to evaluate the migration thresholds for prostheses suspected for loosening. Several studies have shown that the typical migration pattern of an uncemented component differs from that of a primary fixated component, especially during the first 2 years (Nilsson et al. 2006, Dunbar et al. 2009, Pijls et al. 2012a, Wilson et al. 2012, Henricson and Nilsson 2016, van Hamersveld et al. 2017). We therefore question whether the current migration thresholds are justified for uncemented prostheses, especially for designs without biological mediators (e.g., hydroxyapatite or highly porous metal) to enhance bone ingrowth, and can be used to classify such implants being loose in RSA studies with only 2 years of follow-up.

In our study, 1 TKA was revised at 10-year follow-up due to progressive pain and function impairment due to mechanical failure. Posteromedial polyethylene wear and tibial component loosening was found during revision surgery (Figure 5, see Supplementary data). This patient was not flagged as “loose” through RSA measurements as MTPM values were stable up to 5 years of follow-up but further follow-up measurements were unreliable due to high condition numbers (solid red line in Figure 2). The exact failure mechanism is unknown. Causal factors of posteromedial failure include overloading the medial compartment and malalignment of the femoral component, increasing posteromedial peak contact stresses (Morra et al. 2003). Some authors have reported that by cross-linking the polyethylene the fatigue crack propagation resistance is decreased, especially in TKA (Bradford et al. 2004, Ries 2005). However, later reports of fatigue failure are rare and mainly limited to tibial post fractures in posterior-stabilized knees, suggesting this mechanism is unlikely to account for failure in our patient (Jung et al. 2008, Yu et al. 2016).

Although all other subscales of the KOOS score were similar between uncoated and PA-coated components, we did observe a statistically significant difference in the quality of life subscale after 10 years of follow-up. Similar to the occurrence of both the infection cases and the revised case due to mechanical failure (which could all have occurred in either group), the statistical difference in quality of life is most likely a spurious finding and not related to the implant type. Nevertheless, we continue to monitor these patients to observe whether any adverse effect of the given treatment occurs.

Several limitations can be noted. First, a high number of patients were lost to follow-up. Consequently, only 16 patients were available for analysis in the PA-coated group at 10-year follow-up. However, results from the linear mixed-effects model are based on all measurements, not only on remaining patients at final follow-up. Furthermore, as most implants of the lost patients appeared to have stabilized, it is unlikely that the observed results would substantially differ from those presented if patients had continued follow-up. Results of the secondary clinical outcomes should, however, be regarded as exploratory due to the limited sample size and the lower accuracy and precision of these outcome measurements. Second, it remains unknown why 6 components stabilized while 2 did not. Logically, the magnitude of component migration plays a role in preventing the onset of a prosthesis-settling phase. However, other (baseline) factors that may predict high risk patients cannot be found without performing “one-variable-at-a-time” subgroup analyses, which are likely both underpowered and produce false-positive results due to multiple comparisons (Kent et al. 2016). We therefore refrained from performing such subgroup analyses. Third, a strict intention-to-treat analysis requires all randomized patients to be analyzed, which was not the case for the 2 excluded patients on the day of surgery. These 2 patients were excluded from further follow-up measurements at the time, hence no data were available for analysis. Furthermore, not receiving the studied intervention can be a legitimate reason for patient exclusion without risking bias, even in an intention-to-treat trial (Fergusson et al. 2002).

In summary, the typical biphasic migration pattern of uncemented implants was seen in both the uncoated group and the PA-coated group, but the latter showed statistically significantly less mean migration and absence of components with continuous migration at 10-year follow-up. When evaluating uncemented prostheses, especially those without biological mediators to enhance bone ingrowth, the initial migration phase is longer than in cemented components and can last over 2 years. With such prostheses, short-term RSA cut-off values to determine the risk of failure seem of limited value. Evaluation should thus be based on longer follow-up data and include mean migration results as well as individual component migration results. 

### Supplementary data

Tables 3 and 4 and Figures 3–5 are available in the online version of this article, http://dx.doi.org/10.1080/17453674.2018.1469223

The study was designed by STL. Surgeries were performed by STL and 2 other colleagues. Data collection and RSA analysis were performed by KH. Statistical analysis was done by KH and PM. KH, PM, RN, and STL interpreted the data and wrote the initial draft manuscript. KH, PM, RN, and STL critically revised and approved the manuscript.

*Acta* thanks Anders Henricson and Leif Ryd for help with peer review of this study.

## Supplementary Material

IORT_A_1469223_SUPP.pdf
