# Supplementary material for: Peri-apatite coating decreases uncemented tibial component migration: long-term RSA results of a randomized controlled trial and limitations of short-term results
Source: Acta Orthop. 2018 May 9;89(4):425–30. doi: 10.1080/17453674.2018.1469223 (PMC6066768; doi:10.1080/17453674.2018.1469223)
Supplement: IORT_A_1469223_SUPP.pdf [file IORT_A_1469223_SM7226.pdf]

Supplementary data

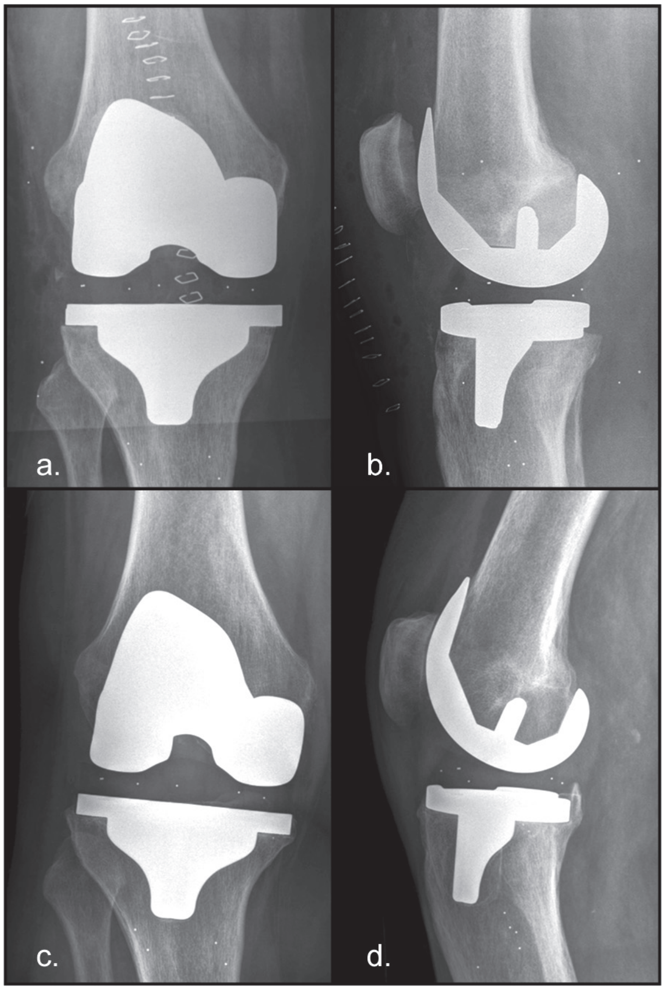

Figure 3. Uncoated TKA in a 65-year-old female (BMI 30) with a preoperatively valgus aligned knee (HKA 186°; postoperative HKA 175°). a, b directly postoperative, c, d at 10-year follow-up. The tibial component was initially classified as loose and did not stabilize between 2 and 10 years: note the posterior tilt of 9° (with a radiolucent line posterior to the tibial keel and posterior subsidence of the tibial plateau in d) and varus tilt of 6° (with medial subsidence of the tibial plateau in c).

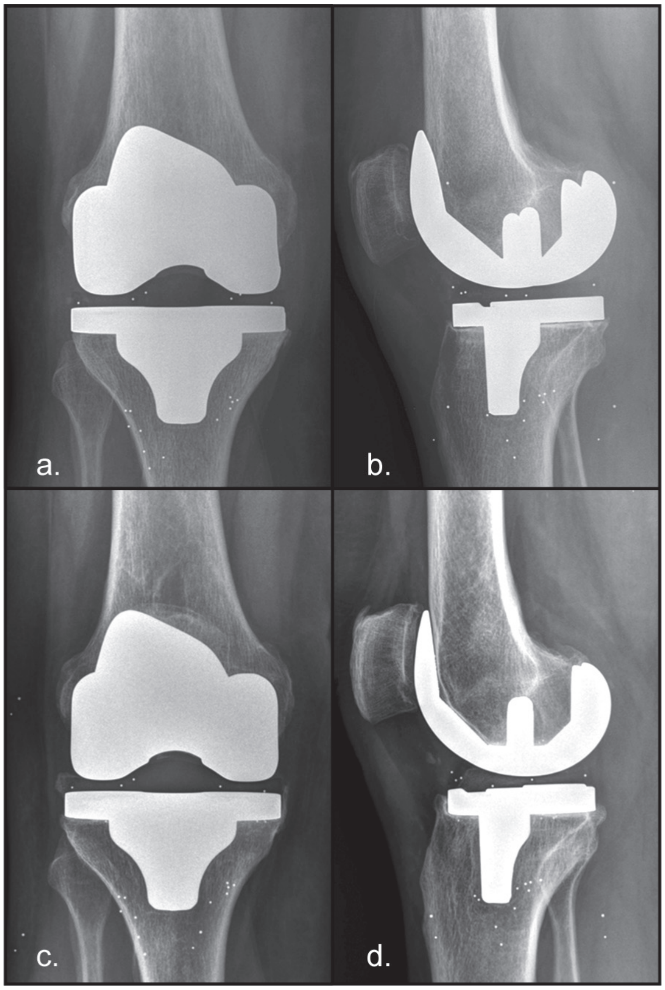

Figure 4. Uncoated TKA in a 56-year-old female (BMI 26) with a preoperatively varus aligned knee (HKA 169°; postoperative HKA 176°). a, b at 3-month follow-up, c, d at 7-year follow-up. The tibial component was initially classified as loose and did not stabilize between 2 and 7 years: note the anterior tilt of 5° (with a radiolucent line anterior to the tibial keel and anterior subsidence of the tibial plateau in d) and varus tilt of 2° (with medial subsidence of the tibial plateau in c).

Table 3. Post hoc analysis of between-group mean difference in logMTPM with different time points as baseline: values are mean (standard error) unless otherwise specified

Baseline	LogMTPM Uncoated	LogMTPM PA-coated	Between-group difference mean (95% CI)	p-value
Postoperative–10 years	0.44 (0.04)	0.29 (0.03)	0.15 (0.08 to 0.22)	< 0.001
3 months–10 years	0.12 (0.02)	0.02 (0.02)	0.09 (0.04 to 0.14)	< 0.001
1 year–10 years	0.03 (0.01)	0.00 (0.01)	0.03 (–0.00 to 0.05)	0.1
2 years–10 years	0.01 (0.01)	0.00 (0.01)	0.01 (–0.02 to 0.03)	0.7

Table 4. Clinical scores in degrees or points (95% CI)

	Preoperatively		5 years		10 years		
	Uncoated	PA-coated	Uncoated	PA-coated	Uncoated	PA-coated	p-value [a]
Knee function (°):							
Flexion	113 (108–118)	118 (112–123)	121 (117–125)	126 (122–130)	123 (120–126)	127 (124–131)	0.9
Extension [b]	–4 (–5 to –2)	–4 (–6 to –3)	–0 (–1 to 1)	0 (–0 to 1)	0 (0 to 0)	0 (0 to 0)	0.5
Knee Society Score (KSS points):							
Knee Score	40 (35–44)	38 (34–43)	97 (92–100)	99 (96–100)	94 (86–100)	97 (94–100)	0.3
Function Score	57 (52–61)	53 (48–58)	88 (81–96)	89 (81–96)	84 (76–91)	86 (78–94)	0.2
Knee injury and Osteoarthritis Outcome Score (KOOS points):							
Symptoms	46 (39–54)	48 (41–55)	91 (85–96)	90 (85–95)	91 (85–96)	90 (85–95)	0.6
Pain	41 (34–48)	41 (34–48)	89 (83–96)	89 (83–96)	91 (86–96)	90 (85–95)	0.7
ADL	44 (36–51)	47 (40–54)	85 (77–93)	87 (80–95)	87 (80–93)	86 (80–93)	0.7
Sports	12 (7–17)	9 (5–14)	45 (33–58)	33 (21–45)	49 (35–63)	54 (39–69)	0.6
Quality of life	23 (18–28)	23 (18–28)	83 (75–92)	84 (75–91)	82 (74–89)	75 (67–83)	0.02

[a] p-values stated in this column indicate testing the between-group mean differences of improvement with time over the entire postoperative follow-up period. Note that not all follow-up measurements are stated, but results from all follow-up measurements were used in the linear mixed-effects model to test for differences.
[b] Negative extension means no full extension possible.

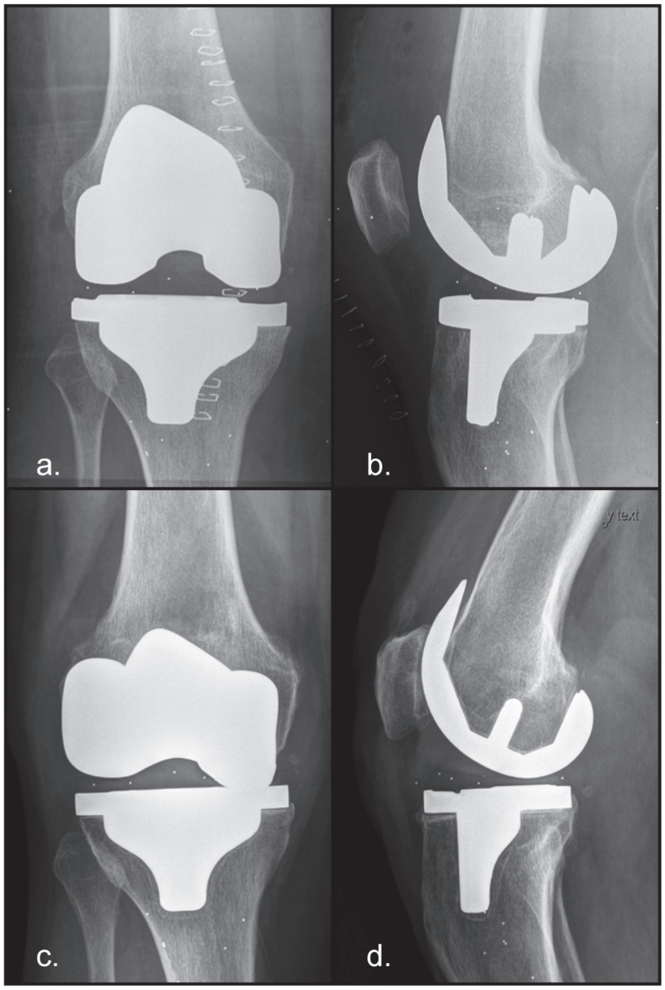

Figure 5. Uncoated TKA in a 60-year-old female (BMI 23) with a preoperatively varus aligned knee (HKA 176°; postoperative HKA 179°). a, b directly postoperative, c, d at 10-year follow-up prior to revision surgery. The tibial component was not classified as loose, which might be due to a different failure mechanism of insert wear, instability, and subsequent loosening. RSA measurements were stable up to 5 years, the measurements at 7- and 10-year follow-up were unreliable (as 2 insert markers were over-projected by the femoral component causing high condition numbers). Note the radiolucent lines around the tibial keel (in both c and d) and anteriorly (in d). A possible defect of the insert was confirmed intraoperatively on the posteromedial side. Contrarily to the well-fixed femoral component, the tibial component was easily extracted.
